# Aging, Gender and Quality of Life (AGEQOL) study: factors associated with good quality of life in older Brazilian community-dwelling adults

**DOI:** 10.1186/s12955-014-0166-4

**Published:** 2014-11-30

**Authors:** Ana Cristina Viana Campos, Efigênia Ferreira e Ferreira, Andréa Maria Duarte Vargas, Cecilia Albala

**Affiliations:** School of Dentistry, Universidade Federal de Minas Gerais, Presidente Antônio Carlos 6627, Belo Horizonte, 31270-901 Minas Gerais Brazil; Department of Community and Preventive Dentistry, School of Dentistry, Universidade Federal de Minas Gerais, Presidente Antônio Carlos 6627, Belo Horizonte, 31270-901 Minas Gerais Brazil; Unidad Nutrición, Salud Pública y Envejecimiento Saludable, INTA, Universidad de Chile, El Líbano 5524 Macul, Santiago, 138-11 Chile

**Keywords:** Quality of life, Aging, Gender

## Abstract

**Background:**

In Brazil, a rapidly aging country suffering from large inequalities, the study of the quality of life (QOL) of aged people is important for the future health. The aim of this study was to examine the associations among QOL, gender, and physical and psychosocial health in older Brazilian community-dwelling adults to identify factors that are associated with better QOL.

**Methods:**

The “Aging, Gender and Quality of Life (AGEQOL)” study, which included 2,052 respondents aged 60 or older, was conducted in Sete Lagoas, Brazil between January and July 2012. The respondents answered questions regarding their socioeconomic and demographic information, health and social situations, cognitive impairment, depressive symptoms and family satisfaction. The authors also applied the Brazilian version the World Health Organization Quality of Life QOL Assessment-Brief Instrument (WHOQOL-BREF) and the World Health Organization Quality of Life Instrument-Older Adults Module (WHOQOL-Old). Ordinal logistic regression with the Proportional-Odds and Logit function was used to test the association between QOL and physical and psychosocial health according to age and socioeconomic status.

**Results:**

Older adults of both genders with five or more years of education, good self-rated health, an absence of depressive symptoms, and no family dysfunction reported better QOL. Retired men had a better QOL compared to non-retired men (OR = 2.2; 95% CI = 1.4–3.2), but this association was not observed in females. Men living in mixed arrangements (OR = 0.5; p = 0.033) and women who did not practice physical activity (OR = 0.7; p = 0.022) tended to have poorer QOL.

**Conclusions:**

We conclude that there are gender differences related to better QOL in this sample. Women with good physical and psychosocial health are more likely to have a better QOL. For men, the best QOL was associated with high socioeconomic conditions and good physical and psychosocial health.

## Background

In Brazil, an increase in life expectancy and a decrease in the fertility rate have led to a significant aging population. In South America, the aging population/proportion of older people is increasing at a more rapid rate than in most developed countries [1,2].

Aging is a complex phenomenon that requires increasing numbers of multidisciplinary studies. The term “active aging”, which was adopted by the World Health Organization (WHO), involves optimizing the opportunities for health, participation and security to improve the quality of life (QOL) as individuals age [3]. The challenge for aging studies is to understand the conditions associated with aging as a positive process and old age as a stage of life in which health, well-being, pleasure and QOL can be increased [4-6].

The QOL of older adults could be good, or at least preserved, provided they have autonomy, independence and good physical health and provided they fulfill social roles, remain active and enjoy a sense of personal meaning [7]. Epidemiological population-based studies are important for identifying the determinants and etiological factors associated with aging. To investigate the determinants of aging, questions must be answered using longitudinal surveys [8]. Longitudinal studies specifically designed to assess health, QOL and associated risk factors are not abundant in the literature, particularly those performed in underdeveloped countries in which poverty and a low educational level might lead to a different set of variables that affect the aging process [9].

In Brazil, a country that is rapidly aging and that suffers from large inequalities, the study of the QOL among aged people is important for the future health. This study sought to examine the association between QOL, gender and physical and psychosocial health among older Brazilian community-dwelling adults, with the aim to identify potential factors associated with better QOL.

## Methods

The Aging, Gender and Quality of Life (AGEQOL) study is an observational, cohort study of a community-dwelling population aged 60 years and older. The sample is representative of the city of Sete Lagoas in the state of Minas Gerais, Brazil, which has a population of approximately 21,000 older adults (10.2% of the population) [10]. This city is divided into 17 administrative regions, one district and four rural areas [11].

### Sample

A complex sampling design was adopted for this study and consisted of a combination of probabilistic sampling methods for selecting a representative sample of the population [12]. For this sampling, the following two calculations were performed: an estimation of the number of older adults and an estimation of the number of households to be visited.

The sample size calculation was performed to compare genders by considering the prevalence of functional impairment in instrumental activities for males (86.6%) and females (72.9%) [13]. The estimated error was up to 5%, with a power of 80% at 95% confidence intervals (95% CI) when considering a design effect of two. An estimated additional 20% of the sample size was added to compensate for refusals. The samples from each group (men and women) were stratified by age in relation to the population and were corrected based on the probability of dying.

Of the total potential participants living in the selected dwellings, 25 (1.2%) were excluded because they could not answer the questionnaire or because of cognitive impairment/dementia or difficulty speaking. One hundred and twenty-five subjects (5.8%) refused to participate in the study, and 100 (4.8%) could not be located or had died. The final sample consisted of 2,052 individuals, of whom 59.7% were female.

The sampling process was conducted in two stages. The census tracts were first selected, and the households within each sector were then selected [10]. In each household, all residents aged 60 years or older were interviewed, regardless of marital status or kinship.

### Data collection

A pilot study including 107 older adults (approximately 10% of the sample) was conducted prior to data collection. All of the instruments were validated for Portuguese in Brazil, and the test/retest method was used to assess reliability and concordance. Coefficients greater than 0.80 were obtained (p < 0.001) and included a weighted Kappa (95%) value of 0.81 (0.71 to 0.91) and an adjusted Kappa value of 0.86.

The data collection was conducted in the homes of the older adults between January and July 2012 and involved household interviews and examinations conducted by three examiners and three annotators.

All persons 60+ years in the selected households were informed of the study and were asked to sign an informed consent form that had been previously approved by the Ethical Committee of the Federal University of Minas Gerais. The interviews lasted 40 to 60 minutes. At the end of the interviews, each subject in the city received guidance regarding health care and activity options as well as the personal contact information of the researcher responsible for the questionnaire.

### Measures

The socioeconomic and demographic data included age, gender, marital status, income categorized by the median value, years of education, residence and occupation. Most independent variables were dichotomized to enhance the interpretability of the logistic regression coefficients.

Physical activity and social participation were measured using a single question with a dichotomous answer (yes or no). The health-related component included self-reported health conditions, which were assessed using a Likert scale, and access to and utilization of health services. For this study, the categories were grouped into poor (very poor and poor), regular and good (good and very good). With regard to the chronic diseases previously reported to be most relevant to the loss of functionality in aging subjects (hypertension, diabetes, cardiovascular disease, musculoskeletal disorders and respiratory diseases), the number of diseases was recorded as 0, 1 or ≥2.

Functional limitations were evaluated by combining the participants’ responses to questions about six basic activities of daily living (eating, dressing and undressing, grooming, walking, getting in and out of bed, bathing and continence) [14] and seven instrumental activities (using the telephone, travel, shopping, meal preparation, housework, taking medicine and management of finances) [15]. To evaluate the cognitive status of the older people, we used the Mini Mental State Examination, which has been validated in Brazil [16] and has a cut-off of 21/22 points [17]. A score ≤21 indicated cognitive impairment.

The presence or absence of a functional limitation was determined depending on the type of daily living activity and cognitive status, as adapted from Albala [18]. The subjects were classified as restricted if they had one or more limitation in basic or instrumental activities or if they had cognitive impairment.

The presence of depressive symptoms was assessed using the short version of the Geriatric Depression Scale (GDS-15) [19], with a cutoff of 5/6; a score ≥6 indicated suspected depression. Family functioning was assessed using the five-item Family Adaptability, Partnership, Growth, Affection, and Resolve (APGAR) scale, which measures the satisfaction of older adults in relation to various aspects of family life [20]. The responses consist of values between 1 (hardly) and 3 (but not always), and the total score ranges from 5 to 15. A score ≥10 indicates family satisfaction [21].

### QOL

We used the World Health Organization Quality of Life Assessment-Brief Instrument (WHOQOL-BREF) [22] and the World Health Organization Quality of Life Instrument-Older Adults Module (WHOQOL-Old) to evaluate QOL [23]. The first instrument is composed of 24 facets that are grouped into four domains that focus on physical, psychological, social and environmental aspects. There is no total score for this instrument, and each item contains five Likert response options that are recorded as scores of 1–5. The WHOQOL-Old module consists of 24 items that are divided into the following six domains: sensory abilities (SAB); autonomy (AUT); past, present and future activities (PPF); social participation (SOP); death and dying (DAD); and intimacy (INT). The scores of all domains are combined to produce an overall score for QOL in older adults, with higher scores indicating good QOL. The instruments were previously validated by Fleck *et al.* [24,25] and showed good reliability and validity in the assessment of QOL of Brazilian older adults (the Cronbach’s alpha score ranged from 0.7 to 0.8 for the WHOQOL-Bref and from 0.7 to 0.9 for the WHOQOL-Old).

### Statistical analysis

SPSS software (SPSS Institute, Chicago, IL, USA) version 19.0 was used for the analysis and included χ^2^ tests and ordinal logistic regression.

**Figure 1:**
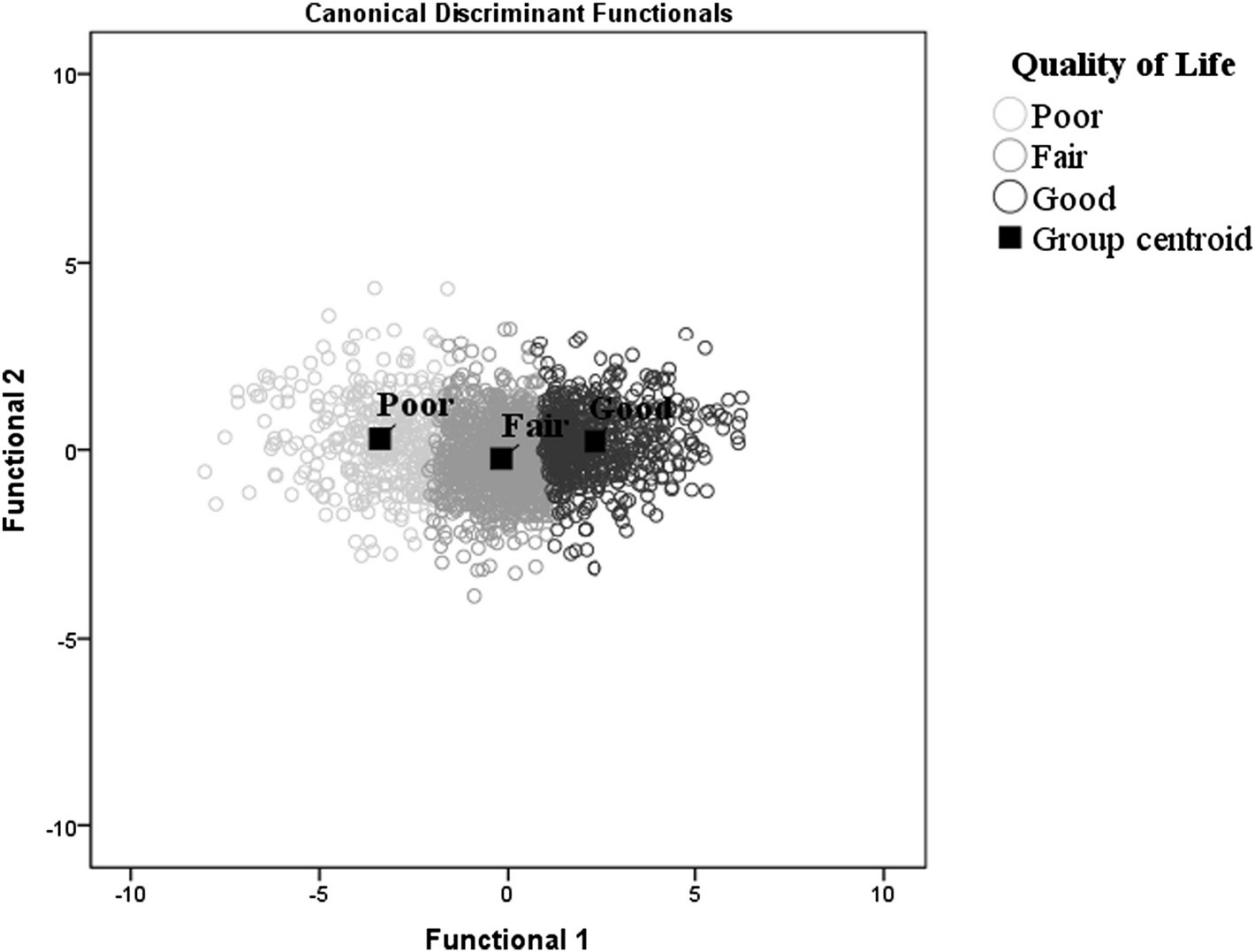
**Canonical discriminant functions based on the QOL level of older people.**

K-means clustering analysis was used to obtain three groups by considering the better distance between the mean scores of the four dimensions in WHOQOL-BREF and the mean of the total WHOQOL-Old score (Figure 1). The F test was used to analyze the differences and characterize the groups with a significance level of 5%. This type of analysis is an analytical statistical tool that is used to define the development of mutually exclusive, significant subgroups based on the similarities among individuals, without prior knowledge of the allocation within the groups. In cases in which the grouping of the data is successful, the groups are internally homogeneous but have high external heterogeneity [26].

Canonical discriminant analysis was used posteriorly to validate the cluster analysis described by two functions. The objective of discrimination is to maximize the variance between and within groups and to verify the efficiency of the overall correct classification of the model [26].

The QOL level among the clusters was adapted from Oliveira *et al.* [27]; for all the WHOQOL domains, there was a group with good QOL scores, a group with intermediate QOL scores and a group with worse QOL scores.

Ordinal logistic regression was used to test the association between QOL and physical and psychosocial health after controlling for age and socioeconomic status. All analyses were performed separately for each gender. In this study, we applied the Polytomous Universal Model (PLUM), which incorporates the ordinal nature of the dependent variable in the analysis; thus, a logistic regression model with proportional-odds and Logit function [28] was performed. The odds between the categories of the dependent variable were compared by calculating the crude and adjusted odds ratio (OR), and tests evaluating the homogeneity of slopes and multicollinearity were conducted using Pearson's adjustment to analyze the validity of the model. To ascertain the possible interference of a small number of observations, we used residual analysis for ordinal data, as proposed by McCullagh [29]. All of these tests showed that the model satisfied all of the assumptions, and the effect of the complex sample design was considered in all of the analyses.

## Results

The age of the total sample at baseline ranged from 60 to 106 years old, and the mean age of all participants at baseline was 70.89 ± 8.14 years (71.03 ± 8.35 for women and 70.69 ± 7.83 for men). Table 1 shows the descriptive statistics of the socioeconomic and health conditions of the participants according to gender. Thirty percent (625) of the participants were more than 74 years old, and 317 (15.4%) older adults were octogenarians. Most men (70.8%) and women (68.7%) were between 60 and 74 years old, and there was no difference in age distribution between genders.

**Table 1:** **Characteristics of the sample by gender (N =2052)**

**Variables**	**Total (n = 2052) n (%)**	**Men (n = 826) n (%)**	**Women (n = 1226) n (%)**	P*
Age				
60-74 years old	1427 (69.5)	585 (70.8)	842 (68.7)	0.162
≥75 years old	625 (30.5)	241 (29.2)	384 (31.3)	
Years of education				
0	579 (28.2)	240 (29.1)	339 (27.7)	0.173
1–4	1282 (62.5)	500 (60.5)	782 (63.8)	
5–7	130 (6.3)	63 (7.6)	67 (5.5)	
>8	61 (3.0)	23 (2.8)	38 (3.1)	
Income**				
≤R$622.00	1357 (66.1)	480 (58.1)	877 (71.5)	<0.001
>R$622.00	695 (33.9)	346 (41.9)	349 (28.5)	
Marital status				
Married	1084 (52.9)	615 (74.5)	469 (38.3)	<0.001
Single/Divorced/Widower	965 (47.1)	211 (25.5)	754 (61.7)	
Living arrangements				
Living with couple	1065 (53.0)	616 (75.5)	449 (37.6)	<0.001
Mixed arrangements	668 (33.2)	121 (14.8)	547 (45.8)	
Living alone	277 (13.8)	79 (9.7)	198 (16.6)	
Functional limitation	754 (36.7)	269 (32.6)	485 (39.6)	0.001
Self-rated health				
Poor	277 (13.5)	105 (12.7)	172 (14.0)	<0.001
Fair	705 (34.4)	242 (29.3)	463 (37.8)	
Good	1070 (52.1)	479 (58.0)	591 (48.2)	
Chronic diseases				
0	327 (15.9)	184 (22.3)	143 (11.7)	<0.001
1	626 (30.5)	274 (33.2)	352 (28.7)	
≥2	1099 (53.6)	368 (44.6)	731 (59.6)	
Physical activity	545 (26.6)	216 (26.2)	329 (26.8)	0.385
Symptoms of depression	619 (30.2)	197 (23.8)	422 (34.4)	<0.001
Cognitive impairment	264 (12.9)	98 (11.9)	166 (13.5)	0.148
Social participation	157 (7.7)	41 (5.0)	116 (9.5)	<0.001
Family satisfaction	1565 (76.3)	630 (76.3)	936 (76.3)	0.520

*χ^2^ test. ** Brazilian minimum salary = R$622.00 ≈ US$300.

Forty-eight types of living arrangements were identified among older adults in the city under study. When taking the three groups of living arrangements that were established in this study into account, it was observed that the majority of older adults who lived alone were women (71.5%), whereas 75.5% of men lived with their partners (p < 0.001). There were no differences in the years of education between the different genders; however, 10.4% of men and 8.6% of women had completed over 4 years of study (Table 1).

Additionally, there were significant differences related to marital status, income, retirement, and living arrangement between genders. The majority of men in the sample were married (74.5%), while 61.7% of women were single, separated or widowed. Most older adults had low monthly income (66.1%), and this percentage was higher for females (71.5%) compared to males (58.1%) (Table 1).

The self-perceived health status was different between men and women (p <0.001). While 50.8% of men rated their health as good, most women rated their health as fair (37.8%) or good (41.8%). Only 15.9% of the older adults did not have chronic diseases; however, the percentage of women (59.6%) with more than two diseases was statistically higher (p < 0.001) than that of men (44.6%). The prevalence of cognitive impairment was 35.3%, with a slightly larger proportion of women (36.0%) than men (34.3%) reporting this condition. In relation to depression, there was a 30.2% prevalence of depressive symptoms and a statistically significant (p < 0.001) difference between genders (23.8% for men and 34.4% for women). There was a high prevalence of functional limitations (36.7%) and a significant difference (p = 0.001) in functional limitations between men (32.6%) and women (39.6%) (Table 1).

Cluster analysis (k-means) resulted in the formation of the following three groups of older adults in relation to QOL (Table 2): subjects with poor, fair and good QOL. The majority of the older adults were included in the fair QOL group (51.4%), which corresponded to the average level of scores in the WHOQOL. The group with worse QOL included 371 people (18.1%), whereas the good QOL group included 627 subjects (30.6%). The results of the test for equality of the group means between the groups were significant, indicating that the groups differed in all QOL domains. The overall correct classification of the canonical discriminant functions was 97.9%, with a correlation coefficient of 0.89.

**Table 2:** **Means and standard deviations of QOL clusters of older adults**

**Domains WHOQOL**	QOL*			**Z**	**Wilks’ lambda test**	**P**
	**Poor (n = 371)**	**Fair (n = 1054)**	**Good (n = 627)**			
**WHOQOL-BREF**	**Mean (±sd)**	**Mean (±sd)**	**Mean (±sd)**			
Physical	41.0 (±14.0)	61.4 (±11.0)	78.3 (±10.1)	1282.4	0.4	<0.001
Psychological	47.7 (±12.4)	68.2 (±9.0)	80.3 (±8.8)	1335.3	0.4	<0.001
Social Relations	52.0 (±16.1)	69.1 (±10.5)	78.8 (±12.5)	555.1	0.6	<0.001
Environmental	47.3 (±10.3)	58.9 (±8.3)	72.9 (±10.5)	913.5	0.5	<0.001
WHOQOL-Old	53.1 (±9.4)	65.4 (±7.7)	77.0 (±8.4)	1007.2	0.5	<0.001

*Final central cluster for QOL measure. Sd = standard deviation.

Differences were observed in all of the QOL variables, except for those of retired individuals. In this case, the socioeconomic distribution between the genders was reversed, with 47.7% of the older adults in the higher QOL group being male and 67.7% of those in the lower QOL group being female. Additionally, there was a gradient association between low QOL and worse health perception, cognitive impairment, depressive symptoms, family dysfunction and functional limitation. Most of the older adults who reported two or more chronic diseases (70.4%) were allocated to the low QOL group (Table 3).

**Table 3:** **Characteristics of the sample by QOL**

**Variables**	**QOL**			P*
	**Poor (n = 371)**	**Fair (n = 1054)**	**Good (n = 627)**	
Age	n (%)	n (%)	n (%)	
60-74 years old	219 (59.0)	744 (70.6)	464 (74.0)	<0.001
≥75 years old	152 (41.0)	310 (29.4)	163 (26.0)	
Gender				
Male	120 (32.3)	407 (38.6)	299 (47.7)	<0.001
Female	251 (67.7)	64.7 (61.4)	328 (52.3)	
Years of study				
0	159 (42.9)	309 (29.3)	111 (17.7)	<0.001
1–4	195 (52.6)	668 (63.4)	419 (66.8)	
>5	17 (4.5)	77(7.3)	97 (15.4)	
Income**				
≤R$622.00	265 (71.4)	730 (69.3)	362 (57.7)	<0.001
>R$622.00	106 (28.6)	324 (30.7)	265 (42.3)	
Retired	266 (71.7)	778 (73.8)	474 (75.6)	0.392
Marital status				
Married	148 (39.9)	554 (52.7)	382 (61.0)	<0.001
Single/Divorced/Widower	223 (23.1)	498 (51.6)	244 (25.3)	
Living arrangements				
Living with spouse	145 (39.9)	544 (52.8)	376 (60.9)	<0.001
Mixed arrangements	162 (44.6)	351 (34.1)	155 (25.1)	
Living alone	56 (15.4)	135 (13.1)	86 (13.9)	
Functional limitation	210 (27.9)	377 (50.0)	167 (22.1)	<0.001
Self-rated health				
Poor	129 (34.8)	113 (10.7)	35 (5.6)	<0.001
Fair	179 (48.2)	407 (38.6)	119 (19.0)	
Good	63 (17.0)	534 (50.7)	473 (75.4)	
Chronic diseases				
0	29 (7.8)	150 (14.2)	148 (23.6)	<0.001
1	81 (21.8)	306 (29.0)	239 (38.1)	
≥2	261 (70.4)	598 (56.7)	240 (38.3)	
Physical activity	59 (15.9)	250 (23.7)	236 (37.6)	<0.001
Cognitive impairment	88 (23.7)	115 (10.9)	61 (9.7)	<0.001
Depressive symptoms	247 (66.6)	300 (28.5)	72 (11.5)	<0.001
Social participation	20 (5.4)	77 (7.3)	60 (9.6)	0.047
Family satisfaction	200 (53.9)	831 (78.8)	534 (85.2)	<0.001

*χ^2^ test. **Brazilian minimum salary = R$622.00 ≈ US$300.

The results of the ordinal regression model, which estimates the OR of good QOL by gender, are shown in Table 4. Age, marital status, income, and cognitive impairment did not remain associated with QOL in the final model.

**Table 4:** **Results of ordinal logistic regression that explain better QOL in older adults, separated by gender**

**Variables**	**Final model**		
**Male**	**OR**	**95% confidence interval**	**P**
Years of education			
>5	4.2	2.4–7.4	<0.001
1–4	2.2	1.3–3.7	0.003
0	1.0		
Living arrangements			
Living with spouse	0.9	0.4–2.0	0.809
Mixed arrangements	0.5	0.3–0.9	0.033
Living alone	1.0		
Retired			
No	0.4	0.3–0.7	<0.001
Yes	1.0		
Self-rated health			
Good	5.7	3.5–9.4	<0.001
Fair	3.0	2.2–4.3	<0.001
Bad	1.0		
Chronic diseases			
>2	0.6	0.4–0.9	<0.001
1	0.6	0.4–0.8	<0.001
0	1.0		
Depressive symptoms			
No	3.6	2.5–5.2	<0.001
Yes	1.0		
Family dysfunction			
No	1.8	1.3–2.6	0.001
Yes	1.0		
**Female**	**OR**	**95% confidence interval**	**P**
Years of education			
>5	2.2	1.3–3.6	<0.001
1–4	1.3	0.9–2.1	0.188
0	1.0		
Self-rated health			
Good	4.2	2.8–6.2	<0.001
Fair	3.0	2.3–4.0	<0.001
Bad	1.0		
Chronic diseases			
>2	0.5	0.3–0.7	<0.001
1	0.7	0.5–0.9	0.010
0	1.0		
Physical activity			
No	0.7	0.5–1.0	0.022
Yes	1.0		
Depressive symptoms			
No	2.2	1.7–2.9	<0.001
Yes	1.0		
Family dysfunction			
No	3.0	2.3–4.0	<0.001
Yes	1.0		

There was an education gradient for the QOL of men. Men with 1–4 and >5 years of education were 2.2 and 4.2 times more likely to have a better QOL than illiterate men. Similarly, women with five or more years of education were associated with good QOL (OR = 2.2; p < 0.001) (Table 4).

Retired men had better QOL when compared to non-retired men (OR = 2.2; 95% CI = 1.4–3.2), but this association was not observed in females. Men living in mixed arrangements (OR = 0.5; p = 0.033) and women who did not practice physical activity (OR = 0.7; p = 0.022) tended to have a poorer QOL (Table 4).

As shown in Table 4, there was an increase in the OR for the association between QOL and self-rated health for both genders once the model was adjusted for demographic variables and psychosocial health. Men with fair health (OR = 3.0; 95% CI = 2.2–4.3) and, in particular, good health (OR = 5.0; 95% CI = 3.5–9.4) were associated with good QOL. Women with good and fair health were 4.2 (OR = 4.2; 95% CI = 2.8–6.2) and 3.0 (OR = 3.0; 95% CI = 2.3–4.0) times more likely to have a good QOL, respectively.

For both genders, there was a robust association between QOL and all psychosocial variables, except cognitive impairment. Men without depressive symptoms and women without family dysfunction were 3.6 (OR = 3.6; 95% CI = 2.5–5.2) and 3.0 (OR = 3.0; 95% CI = 2.3–4.0) times more likely to have good QOL, respectively (Table 4).

## Discussion

The physical and psychosocial health and socio-demographic variables examined in this study were evaluated using ordinal logistic regression, which resulted in the following five variables being associated with good QOL for both genders: self-rated health, depressive symptoms, years of education, chronic diseases, and family dysfunction. Additionally, good QOL for men was associated with retirement, mixed living arrangements, and physical activity, whereas good QOL for women was associated with physical activity; these results are similar to those of other studies [27,30,31]. These factors represent targets for policy action because they have the potential to affect the health of older individuals in the general population.

A number of studies have been performed on QOL in older adults. This study is original and innovative because it used a representative sample to provide information regarding an ordinal positive relationship between QOL and self-rated health. Furthermore, our results indicate that the most important factors for a good QOL for both genders is a good health perception and a lack of depression, even when the model was adjusted for socioeconomic conditions.

We observed a significant difference of 4.4% when comparing good self-rated health between the low and high QOL groups. In the ordinal regression, the men and women who reported having good health were 5.7 and 4.2 times more likely to have good QOL, respectively.

Previous studies on QOL in older adults have also shown a direct relationship with self-rated health [27,32,33]. In particular, older adults who evaluated themselves as having good health tended to have good QOL [27].

The perception of health in older adults was generally positive because most of the older adults in this sample rated their health as good (52.1%), including 58.0% of the men and 48.2% of the women (48.2%). However, the percentage of poorer self-rated health was higher in women compared to men. This study provides further evidence that QOL can be explained by self-rated health and its associated factors among older men and women.

In the SABE study (Salud, Bienestar y Envejecimiento) in São Paulo, Brazil, 8.9% of women and 7.2% of men demonstrated poor health. In other SABE study countries, the participants reporting good/very good health ranged from 27.9% of women (Mexico) to 69.0% of men (Uruguay) [2].

A previous study on the components of self-rated health among adults suggested that physical health (chronic diseases and functional limitations) most likely comprises the majority of an individual’s perception of health status [34], and this result was observed in this study.

Health perception involves an individual’s evaluation of his/her body in relation to his/her feelings, including feelings regarding health and well-being, and this perception can be altered by environmental stressors and the social context [35]. For older adults, the concept of self-rated health remains stable despite significant health problems, although over time, there might be a reduction in the standard of good self-rated health [36].

Self-rated health has been shown to be a reliable method for measuring health status [37] and to be a consistent predictor of mortality in older adults [38]. It is essential to use the association between perceived health and QOL in patients, especially in regards to the dual direction of this association.

We observed a very strong association between QOL and depressive symptoms, which corroborates the findings of other studies [32,33,39,40]. Thus, the choice of good QOL was 3.6 and 2.2 times higher for men and women without depressive symptoms than for those experiencing depressive symptoms, respectively. This finding could be explained by the high prevalence of depression (30.2%) in this sample; this figure reached 34.4% in women and 70.4% in women with poor QOL. These disorders are more prevalent in females, but this gender vulnerability varies with age [41].

In a study conducted in Łódź, Poland, 30.9% of older adults (56.5% females) were found to suffer from depression. According to the authors, the chances of good self-rated QOL were 9.9 (95% CI = 5.0–19.6) times higher in older adults without depression [42].

Considering the importance of the physical and psychosocial aspects of active aging and of QOL in older adults, other results of this study should be briefly discussed. In this study, an increase in the number of chronic diseases was associated with a decrease in QOL, and statistically significant gender differences were observed between chronic diseases and QOL. Most women who reported two or more diseases were classified as having poor (73.3%) or fair (62.3%) QOL (data not shown). In general, the prevalence of chronic disease among older people in Brazil is high and differs between genders [11], resulting in negative repercussions on QOL [43]. Preventive actions and the promotion of policies for controlling the effect on health conditions could result in good QOL in this population [30].

Physical activity is a protective factor for QOL and has been previously discussed in the literature [44,45]. For women, we observed a significant association between QOL and physical activity (OR = 0.7; 95% CI = 0.5–1.0), i.e., the choice of good QOL was 1.4 times higher for women who practiced physical activity. However, this association was not accurate once the confidence intervals included a value of 1.0. Physical activity was measured using a single yes/no question, which is an important limitation of this study because these results assume that any level of physical activity will be associated with health.

We did not observe an association between marital status and QOL, although we observed an inverse association between QOL and family dysfunction. Men and women who were satisfied with their family relationship had 1.8 and 3.0 times higher odds of good QOL, respectively. Frequent contacts and visits with friends or family have been shown to motivate activity and increase self-rated QOL [46].

Additionally, we found a high percentage of individuals with poor QOL living in mixed arrangements, i.e., sharing the household with their sons and frequently with sons and grandchildren (44.6%). This situation, which is common in other Brazilian regions, is in contrast to the living arrangements in developed countries [11].

As shown in Table 4, men living in mixed arrangements had worse QOL than those living alone. In our dataset, most men who lived in mixed arrangements had functional limitations and reported more than two chronic diseases (63.3%). It is possible that men in our sample could have been living in mixed arrangements because they had poorer health and therefore needed daily assistance; however, these results should be interpreted with caution, as there was a low percentage of men living alone (9.7%). Size, sample stratification and corrections minimized these effects, thus permitting comparisons in this study.

A mixed living arrangement could have a negative effect on the older population [20,47]. However, living alone presents a greater risk of loneliness and isolation because loneliness increases as the social contacts of older individuals decrease [46].

Similar to the results of other studies [33,48,49], we found an association between QOL and education. Sete Lagoas is a Brazilian city with high life expectancy (73.9 years) and good social indicators [50]. In addition, illiteracy is high in this sample (28.2%) compared to the current national data (24%) [51]. These results are often found in most Latin American countries [2] and in some regions in Brazil, where very different educational opportunities are available for the rich and poor.

A low level of education is an important aspect to be considered when developing public policies for older adults and a proposed collective action. In our study, the illiteracy rate was similar between genders (29.1% for men and 27.7% for women). A previous study investigated trends in educational inequalities in terms of old-age mortality in Norway from 1961 to 2009, and the authors observed that relative educational inequalities in old-age mortality were increased for both genders [52].

The association of years of education with QOL was different between the genders. We observed an ordinal crescent impact of years of education on QOL for men, indicating that education can be a protective factor for good QOL among men. The QOL among women with 1–4 years of education was no different than that of illiterate women.

Our results correspond to the baseline data reported for the AGEQOL study. However, the lack of understanding of the ways in which specific levels of education interfere in the association between SES and QOL is the first limitation of this study. A longitudinal follow-up study of older adults would permit better comparisons of this study with others, although such comparisons might be hampered by differences in the QOL models and measures that are employed across studies. It is not yet possible to determine whether there is a temporal relationship between the studied variables.

The response rate in this study could be considered high (98.8%); therefore, this study is one of the few studies that have been performed using a probabilistic sample of older adult community residents with an adequate number of participants to perform an ordinal logistic regression. Our results are valid and representative of the population living in the community that lacks significant cognitive and/or physical deficits.

In addition to the limitations of this being a cross-sectional study, it should be emphasized that the evaluation of QOL presupposes the quantification of a construct that is sensibly marked by the subjectivity of individual experiences, beliefs, expectations and perceptions [24].

In this sense, it is necessary to discuss the instruments used to measure QOL in older adults. We used the WHOQOL-BREF and WHOQOL-Old, which were developed by the WHO, are widely reported in the scientific literature and have been validated in Brazil [24,25]. The results of this study corroborate those reported by the Brazilian WHOQOL group. For older Brazilian adults, a positive QOL includes several aspects such as activity, income, social life and family relationships, whereas a negative QOL is related to poor health, which differs between individuals [53].

WHOQOL-Old is a supplementary module for older adults and can be added to the existing WHOQOL instruments [22]. Bowling [7] compared generic QOL scales used for older adults and showed that the WHOQOL-Old was the most comprehensive instrument; the questions in this instrument are based on measuring suffering, but the questionnaire is relatively long, and the Likert scale format might be boring to the subjects (although there is no evidence that this characteristic has adversely affected responses to date).

Additionally, Bowling emphasized the need for a generic, truly multidimensional QOL measure with minimal respondent burden for evaluating the outcomes of health and social care in older populations [7]. The reason for the existing difficulties in the assessment of QOL that limits its inclusion in clinical practice and public health services is relevant [54].

To minimize these limitations, one specific method of analysis was conducted in this study. Based on a Brazilian study [27], we used cluster analyses and canonical discriminant analyses to compile both WHOQOL instruments into a unique measure for QOL. This analysis was performed to provide an ordinal variable with three internally more homogeneous groups that were distinct from each other. Additionally, we minimized the variations between the mean scores of the five dimensions of QOL that were considered. We found a high percentage of correct classification (97.9%) and a high correlation coefficient (0.89), which indicated the likelihood that we had constructed a good measure of QOL for older adults in this sample.

In future studies, we suggest replicating this statistical model, considering gender and age stratification variations and including other independent variables concerning nutrition and lifestyle. Adaptation and resilience might also play a role in maintaining good QOL [55].

Despite these limitations, this study confirmed that the QOL of older adults differed between the three clusters that were formed, with a good QOL being strongly associated with good self-rated health, the absence of depressive symptoms, and family satisfaction.

Overall, the results demonstrate that active aging in Sete Lagoas, Brazil, does not occur evenly across genders. Better healthcare requires the inclusion of such differences as part of the comprehensive evaluation of older adults [56].

The discussions of aging in the different genders in relation to living conditions and perceived health that are presented in this study need to be further explored, as there are particularities of each group that may have been missed during routine analysis. We believe that this study may contribute to the formulation of new public health and social care policies for older adults in the medium and long term. Older adults will benefit from interdisciplinary monitoring that focuses on promoting health, improving QOL and active aging.

## Conclusions

We conclude that there are gender differences related to better QOL in this sample cohort. Women with good physical and psychosocial health are more likely to have a better QOL. For men, the best QOL was associated with high socioeconomic conditions and good physical and psychosocial health. We hope that our study contributes to future discussions on the most important predictors for assessing QOL in older adults and on long-term changes in the perception of QOL in this population.

## Abbreviations

ADL: Activity of daily living
APGAR: Adaptability, partnership, growth, affection, and resolve
AUT: Autonomy
DAD: Death and dying
GDS: Geriatric depression scale
IADL: Instrumental activities of daily living
95% CI: 95% confidence interval
INT: Intimacy
MMSE: Mini-mental state examination
OR: Odds ratio
PPF: Past, present and future activities
PLUM: Polytomous universal model
QOL: Quality of life
SAB: Sensory abilities
SOP: Social participation
WHO: World Health Organization
WHOQOL-BREF: World Health Organization Quality of Life Assessment-Brief Instrument
WHOQOL-Old: World Health Organization Quality of Life Instrument-Old
